# How to define core entrustable professional activities for entry into residency?

**DOI:** 10.1186/s12909-018-1159-5

**Published:** 2018-05-02

**Authors:** Ylva Holzhausen, Asja Maaz, Anna Renz, Josefin Bosch, Harm Peters

**Affiliations:** Dieter Scheffner Center for Medical Education and Educational Research, Dean’s Office for Student Affairs, PDL, Charité – Universitätsmedizin Berlin, Berlin, Germany

**Keywords:** Entrustable Professional Activities, Curriculum development, Undergraduate medical education, Consensus methods

## Abstract

**Background:**

Institutions considering to employ core Entrustable Professional Activities (EPAs) for entry into postgraduate training as outcomes for their undergraduate medical programs can partly build on published examples, but also have to undergo their own content validation process to take their specific context into consideration. This process involves several challenges and is not well-described in the literature. Here, we report in detail on a systematic, literature-based approach we recently utilised at our institution to define core EPAs for entry into residency.

**Main body:**

Central to the process was a modified Delphi consent procedure. It involved a multistep interaction between a writing team and a multidisciplinary panel of experienced physicians. Panel members provided both quantitative ratings and qualitative feedback on the EPA categories title, specification/limitations, conditions and implications of entrustment decision, knowledge, skills, and attitude. Consent was achieved when a Content Validity Index (CVI) of ≥80% was reached. The writing team adjusted the EPA category descriptions on the basis of panel members´ ratings and comments, and specified the EPA categories’ link to competencies and assessment sources. This process produced a description and definition of a full set of core EPAs for entry into residency adapted to our context.

**Conclusions:**

This process description for locally adapted core EPAs for entry into residency may support and guide other medical schools in the development and implementation of EPAs into their programs.

## Background

The definition of core Entrustable Professional Activities (EPAs) for entry into postgraduate training has become an active field of development. Many institutions are currently considering the use of EPAs as outcomes for their undergraduate medical programs [1]. These institutions can build in part on EPAs which have been reported at a national level [2–4] and at a local level [5], but will be required to undertake their own content validation process to adapt these EPAs to their specific context. However, available reports do not include a fully detailed description of the EPA development process which could guide other institutions. In this article, we report in detail on a systematic, literature-based approach we employed to define core EPAs for entry into residency as outcomes for the undergraduate medical curriculum at Charité - Universitaetsmedizin Berlin, Germany (Charité).

**Fig. 1:**
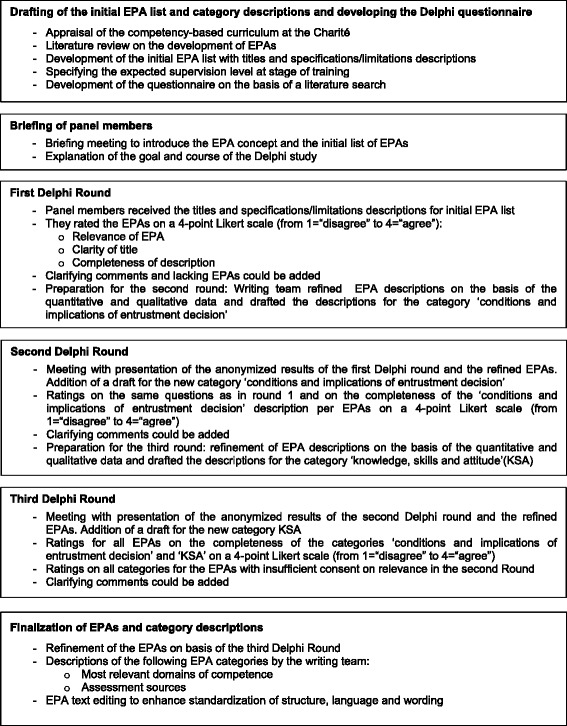
Course of the Delphi Study

We chose a modified Delphi study procedure, an established method for anonymised, non-hierarchical content validation, including EPA development in medical education [1, 6]. As a modification of the Delphi process, panel members received a predefined list of EPAs in the first round. Our goal was the definition of a full set of core EPAs with a seven-category description for each EPA according to current recommendations in the literature (1, 7, 8]. The definition of educational outcomes by EPAs is generally achieved in an iterative process, beginning with the identification of authentic professional tasks, followed by the elaboration of its characteristics, and finally validation of the content by a group of experts [1, 7]. Figure 1 provides an overview of our Delphi study process which involved a multistep interaction between a writing team of educationalists and a panel of experienced physicians.

### Delphi study process

#### Panel selection and writing team

A total of 45 panel members were purposely selected from the Charité faculty body. All panel members had long-time supervision experience in both undergraduate and postgraduate medical training and were actively involved in the curriculum development process for the current undergraduate program. The EPA writing team, consisting of the authors of this article, were members of both the curriculum development group for the undergraduate medical program and educational researchers in the field of EPAs.

#### Guiding principles for EPA content definition

The following guiding principles were formulated for EPA content definition: 1) They should comply with the recommendations for EPA definition [8], i.e. represent independently executable tasks which are observable, measurable, confined to qualified personal and suitable for an entrustment decision. 2) The EPAs should consist of full, seven-category descriptions, including the following categories: ‘title’, ‘specification/limitations’, ‘knowledge, skills and attitudes’ (KSA), ‘conditions and implications of entrustment decision’, ‘most relevant domains of competence’, ‘assessment sources’, and the ‘expected supervision level at the stage of training’. 3) The EPA content elaboration should use clear language describing tasks and workplace context and avoid educational jargon. This includes a short brief title, succinct descriptions as well as an alignment of structure, language and wording within the set of EPAs. 4) The EPAs for entry into residency constitute the core, this is the full set of professional activities expected from a graduating physician. 5) The breadth and level of difficulty of the EPAs should be manageable for graduating physicians, align with the workflow and the supervision routines in the clinical setting, and 6) the supervision level is defined by the time it takes for the supervisor to be physical available as well as the degree of subsequent work verification.

#### Drafting of the initial EPA list and category description

In an iterative process, the writing team drafted the initial list of tasks to be considered as EPAs for entry into residency according to the specific context. This involved mapping these tasks to the Charité competency framework, a search and appraisal of the literature, along with continuous discussions and developments within the Charité curriculum development group. The AAMC core EPAs were used as a starting point [2]. In addition, the writing team consulted articles on the EPAs concept in general [8–12] and articles covering the development of EPAs for postgraduate training [13–16]. The draft of the initial EPA list included tasks which graduating physicians should be able to perform under a granular operationalised level of supervision [17]. The following categories were elaborated for each EPA: ‘title’, ‘specification/limitations’, and ‘expected supervision level at stage of training’. These categories are thought of as those representing the quintessence of an EPA description, upon which the other categories subsequently build.

#### Questionnaire development

The writing team developed the questionnaire for the Delphi process based on the literature on EPA development. For EPA identification and content validation, panel members were asked to rate the relevance of professional tasks for new residents, the clarity of each EPA title and the completeness of the EPA category descriptions on a 4-point scale. The questionnaire was administered online using EvaSys (Electric Paper Evaluationssysteme GmbH, Lüneburg, Germany), a software for survey based-research.

#### Establishing consensus among panellists’ ratings

Content validity indices (CVI) were calculated to establish consensus among the ratings of panel members [18].This included the relevance ratings of the EPAs, the ratings of the ‘clarity of the title’ and the completeness of the EPA categories ‘specification/limitations’, ‘conditions and implications of entrustment decision’ and ‘KSA’. The CVI describes the percentage of respondents who rated the relevance of the EPAs or the categories with ‘agree’ or ‘somewhat agree’. A CVI of at least 80% was set as the predefined consensus level. If this level was reached, consensus was assumed and no further validation was deemed necessary.

#### Panel member invitation and briefing

The panel members were invited to a formal meeting at the beginning of the Delphi study to prepare them for their participation. During the meeting, they were informed about the EPA concept and the aim and structure of the Delphi process. Similar panel meetings were held again before the second and third Delphi rounds. Here, panel members were provided with an anonymised summary of the previous round’s results, the refined EPA content descriptions, and information on subsequent tasks. The meetings were audio-recorded, screen-casted, and sent out to panel members as podcasts along with other material shown in the panel meeting.

##### Round 1

Panel members received the initial draft of EPAs relevant for entering residency including titles and specification/limitations. Panel members provided ratings and could add narrative text for explanations or suggestions for refinement. They were also asked to propose relevant tasks which they felt were missing for entry into residency. The EPA writing team summarised the quantitative and qualitative information provided and refined the EPAs accordingly. The qualitative feedback was clustered inductively and allocated to the corresponding EPA text passages. The proposed changes were then discussed within the writing team until a consensus was reached on the EPA description refinement. The topics for additional EPAs were discussed within the writing team and reviewed on the basis of the above-described guiding principles for EPA content definition.

##### Round 2

Panel members received the anonymised, summarised panel rating results of the first round along with the refined EPA titles and specification/limitations descriptions. Changes made following the first round were highlighted. The panel members received the same questions as in Delphi Round 1. In addition, they were asked to rate a description drafted by the writing team on the EPA category ‘conditions and implication of entrustment decision’ which specifies how the supervision level is operationalised into the workplace. Again, all quantitative ratings could be supplemented with narrative feedback. The EPA writing team summarised the quantitative and qualitative information and adjusted the EPA descriptions as described above for Round 1.

##### Round 3

The panel members were given the anonymised, summarised panel rating results of Round 2. They also received the refined EPA titles and descriptions of the categories ‘specification/limitations’ and ‘conditions and implications of entrustment decision’ with an indication of changes made following feedback in the previous round. Panel members were asked to rate again on the content of the refined categories in those EPAs which had not received sufficient consensus on the relevance rating in the previous round. For the third Delphi round, the writing team drafted the EPA category ‘KSA’ for each EPA. The panel members rated the completeness of the categories ‘conditions and implication of entrustment decision’ and ‘KSA’ in all EPAs. The ratings could be supplemented by narrative comments. In the final round, a CVI of over 80% was reached in the panellists’ ratings on the EPA category descriptions.

#### Finalisation of EPA list and category descriptions

The writing team made final changes to the content of the EPA categories on the basis of panel member ratings and comments from Round 3. EPA categories ‘most relevant domains of competence’ and ‘assessment sources’ were defined in an iterative consensus process with the Charité curriculum development group. Furthermore, special attention was paid to harmonising structure, language and wording in the EPA descriptions.

## Conclusions

This article reports in detail on the process of defining a full set of core EPAs for entry into residency. Our process description may provide support and guidance to other medical schools for the development and implementation of EPAs for their own programs according to their specific contexts.

## Abbreviations

CVI: Content validity indices
EPA: Entrustable Professional Activity
KSA: Knowledge, skills and attitudes
